# Altered dynamics between neural systems sub-serving decisions for unhealthy food

**DOI:** 10.3389/fnins.2014.00350

**Published:** 2014-11-04

**Authors:** Qinghua He, Lin Xiao, Gui Xue, Savio Wong, Susan L. Ames, Bin Xie, Antoine Bechara

**Affiliations:** ^1^Faculty of Psychology, Southwest UniversityChongqing, China; ^2^Department of Psychology and Brain and Creativity Institute, University of Southern CaliforniaLos Angeles, CA, USA; ^3^National Key Laboratory of Cognitive Neuroscience and Learning, IDG/McGovern Institute for Brain Research, Beijing Normal UniversityBeijing, China; ^4^Department of Special Education and Counselling, The Hong Kong Institute of EducationHong Kong, China; ^5^School of Community and Global Health, Claremont Graduate UniversityClaremont, CA, USA

**Keywords:** Iowa Gambling Task (IGT), food choice, Self-Control, Eating, insula

## Abstract

Using BOLD functional magnetic resonance imaging (fMRI) techniques, we examined the relationships between activities in the neural systems elicited by the decision stage of the Iowa Gambling Task (IGT), and food choices of either vegetables or snacks high in fat and sugar. Twenty-three healthy normal weight adolescents and young adults, ranging in age from 14 to 21, were studied. Neural systems implicated in decision-making and inhibitory control were engaged by having participants perform the IGT during fMRI scanning. The Youth/Adolescent Questionnaire, a food frequency questionnaire, was used to obtain daily food choices. Higher consumption of vegetables correlated with higher activity in prefrontal cortical regions, namely the left superior frontal gyrus (SFG), and lower activity in sub-cortical regions, namely the right insular cortex. In contrast, higher consumption of fatty and sugary snacks correlated with lower activity in the prefrontal regions, combined with higher activity in the sub-cortical, insular cortex. These results provide preliminary support for our hypotheses that unhealthy food choices in real life are reflected by neuronal changes in key neural systems involved in habits, decision-making and self-control processes. These findings have implications for the creation of decision-making based intervention strategies that promote healthier eating.

## Introduction

With an increase of abundant and easily accessible high-calorie foods, an important characteristic of human choices in food is the unhealthy consumption of high calorie foods. Such choices can have long-term negative consequences, such as medical problems associated with overweight and obesity. The question is: why do some individuals become insensitive to the future consequences of their unhealthy eating habits and have difficulty making better healthful choices? While some research has found that poorer decision-making capacity may be associated with abnormal eating behaviors, most of these studies have focused on patients with differing forms of eating pathology (Pignatti et al., 2006; Brogan et al., 2010; Danner et al., 2012; Fagundo et al., 2012). In the current study, we evaluate normal individuals who are not medically diagnosed with an eating disorder. We examine the activity of neural systems hypothesized to subserve decision-making, using the Iowa Gabling Task (IGT), as well as the relationship between this neural activity and real life eating behavior.

Recent work has hypothesized that at least three neural systems influence behaviors involving complex decision-making, especially choices that include conflicts between immediate and long-term consequences (Naqvi and Bechara, 2009; Noel et al., 2013; He et al., 2014a,b). One neural system is thought to mediate habitual behaviors that are elicited spontaneously or automatically. This neural system has been referred to as the “Impulsive System,” and key neural regions in this (impulsive) system include the amygdala and ventral striatum (and its mesolimbic dopamine link), which has been found to play a key role in the incentive motivational effects of a variety of non-natural rewards (e.g., psychoactive drugs) and natural rewards (e.g., food) (Stewart et al., 1984; Robbins et al., 1989; Wise and Rompre, 1989; Robinson and Berridge, 1993; Di Chiara et al., 1999; Everitt et al., 1999; Balleine and Dickinson, 2000; Koob and Le Moal, 2001; Dagher, 2009; Dagher and Robbins, 2009). Another neural system relates to executive and inhibitory control, referred to as the “Reflective System,” and a critical neural region in the reflective system is the ventromedial prefrontal cortex (VMPFC) region, as well as the medial orbitofrontal cortex (Bechara et al., 2000). However, other neural components, including the dorsolateral prefrontal cortex implicated in working memory capacity and the cingulate cortex are also parts of this neural circuitry, and are essential for the normal operation of the VMPFC (Bechara, 2004; Boorman et al., 2013).

More recent evidence suggests that there is a third neural system mediated through the insular cortex. This pathway plays a key role in translating interoceptive signals into what one subjectively experiences as a feeling of desire, anticipation, or urge (Naqvi et al., 2007; Naqvi and Bechara, 2009). There is evidence demonstrating that the insular cortex is implicated in drug craving (Garavan, 2010). For example, strokes that damage this region eliminate the urge to smoke in individuals previously addicted to cigarette smoking (Naqvi et al., 2007). Additionally, an increasing number of studies suggest that the insula shows exaggerated responsiveness to drug cues in individuals addicted to drugs, and is hyper-reactive to visual food cues in obese individuals (Killgore et al., 2003; DelParigi et al., 2006; Geliebter et al., 2006; Grill et al., 2007; Rothemund et al., 2007; Stoeckel et al., 2008; Brooks et al., 2013; García-García et al., 2013; Tomasi and Volkow, 2013). Finally, a behavioral measure of urgency, defined as an individual's tendency to give in to strong impulses, specifically when accompanied by negative emotions such as depression, anxiety, or anger (Whiteside and Lynam, 2001), has also been shown to positively correlate with insula activity in recent fMRI studies (Joseph et al., 2009; Xue et al., 2010).

Emerging evidence suggests that overweight and obesity represents a special case of addictive behavior, which involves underlying neural mechanisms similar to other addictions (Kelley and Berridge, 2002; Rolls, 2007; Trinko et al., 2007; Volkow et al., 2008; Johnson and Kenny, 2010). Specifically, a hyper-functioning impulsive system, a hypo-functioning reflective system, and/or an altered insula system were suggested by previous empirical studies as potential candidate mechanisms for the over-eating behavior (He et al., 2014a,b), thus consistent with proposed theories on behavioral addiction to substances in general (Bechara and Damasio, 2005; Naqvi and Bechara, 2009; Noel et al., 2013). Based on these findings, we hypothesized that a loss of self-control or inability to resist tempting/rewarding foods, and the development of less healthful eating habits (e.g., greater intake of high-calorie foods), may be explained by some alternation in any of these three neural systems.

The aim of this study was to utilize a laboratory-based task that taps into the functions of the different neural systems involved in affective decision-making, and to use functional imaging to evaluate the activities of these neural systems in relation to food choices in real-life. The most frequently used paradigm to assess affective decision-making is the Iowa Gambling Task (IGT) (Bechara et al., 1994; Bechara and Damasio, 2002; Waters-Wood et al., 2012), which was initially developed to investigate decision-making defects of patients with focal brain lesions. The IGT has been shown to tap into aspects of decision-making that are influenced by affect and emotion (Bechara and Damasio, 2005). Many studies have demonstrated that in comparison to normal controls, a wide range of patients (e.g., substance users, schizophrenia, pathological gamblers, and adolescents with externalizing behavior) show poor behavioral decisions as measured by the IGT (Bechara and Damasio, 2002; Cavedini et al., 2002; Whitney et al., 2004; Sevy et al., 2007; Xiao et al., 2009). The same set of brain regions (i.e., ventral striatum, prefrontal cortex, and insula) linked to decision-making impairments in brain lesion studies have also been shown to be engaged during functional neuroimaging studies in healthy individuals during performance of the IGT (Li et al., 2010; Xiao et al., 2013).

The present study used Functional Magnetic Resonance Imaging (fMRI) techniques to investigate the relationship between the brain activity underlying decision-making (as elicited by the IGT) and real-life food choices in a group of normal young adults. Specifically, we tested the hypothesis that decision-making during the IGT will activate a neural circuitry that includes the mesial orbitofrontal and VMPFC region, the dorsolateral prefrontal cortex, and the anterior cingulate/SMA (supplementary motor area), which are components of the so-called “reflective system.” The degree of activity in these neural regions was hypothesized to inversely correlate with the degree of self-reported consumption of snacks high in fat and sugar, i.e., higher snack consumption would be associated with lower neural activity. Further, the degree of activity in these neural regions was hypothesized to positively correlate with the degree of self-reported consumption of vegetables, i.e., higher consumption would be associated with higher neural activity. We also tested the hypothesis that decision-making during the IGT would activate a subcortical neural circuitry that includes neural components of the so-called impulsive and urge system, namely the amygdala, the ventral striatum, and the insular cortex. The degree of activity in these neural regions was hypothesized to positively correlate with the degree of self-reported consumption of snacks high in fat and sugar but negatively correlate with the degree of self-reported consumption of vegetables.

## Methods

### Participants

Twenty-three (12 female) healthy adolescents and young adults aged 18.01 ± 2.61 years were recruited from the University of Southern California (USC) and recreation centers in Los Angeles, California. None of the participants were currently diagnosed with an eating disorder or receiving clinical treatment for obesity. All participants had normal or corrected-to-normal vision. Based on the Structured Clinical Interview for DSM-IV (SCID), all participants were free of neurological or psychiatric history. Adolescents who were under 18 were accompanied to the university by a parent or designated family member. Written informed consents were obtained from the participants and their parent/legal guardians (for participants under 18) prior to participation. Research protocols and instruments were approved by the USC Institutional Review Boards.

### Procedures

Participants came to the lab for two sessions. During the first session, participants and their parent (for participants under 18) completed and signed the consent form(s) and completed behavioral tasks. During the second session, participants were returned for the fMRI scan session. We asked participants to have their usual meal before they arrived for the fMRI session and eat normally. Therefore, the last meal was roughly equivalent across all the participants. We measured height and weight of participants using standard procedures. We also assessed the hunger level on a scale ranging from 1 (not hungry at all) to 10 (very hungry) and assure the participants were not in a deprived state prior to the fMRI scan.

### Behavioral tests

Wechsler Abbreviated Scale of Intelligence [WASI, (Wechsler, 1999)]. The WASI was used to measure a participant's Intelligence Quotient (IQ) and basic aspects of cognitive functioning. The WASI is designed for use with a broad age range (from 6 to 89 years of age), is nationally standardized and, similar to other Wechsler scales. It consists of four subtests (Vocabulary, Similarities, Block Design and Matrix Reasoning) chosen based on the high loadings on general intellectual ability (g) and the cognitive skills tapped by each. A combination of the four subtests yields a Full Scale IQ score.

Youth/Adolescent Eating Questionnaire (YAQ) (Rockett et al., 1995). We used the YAQ to assess eating behavior in real life. The YAQ is a self-report food frequency questionnaire with acceptable validity and reliability (Rockett et al., 1995, 1997). It asks about intake of 132 food items over the past year and food items can be grouped for analysis (Xie et al., 2003; Field et al., 2004). In the present study, we were mainly interested in snack and vegetable food consumption. The YAQ includes 25 questions assessing intake of snack foods. Snack items included the items high in sugar (e.g., fruit rollups, Pop-tarts) and those high in fat/high salt (e.g., potato chips, crackers). Reported consumption to these items was summed to calculate daily servings according to previous studies (Field et al., 2004; Xie et al., 2003). The same calculation was done for vegetable items (e.g., celery, carrot).

### fMRI tasks

Participants were scanned while performing an event-related IGT. As described in previous studies (Bechara et al., 1994, 1999), the IGT is a computerized version of a gambling task with an automated and computerized method for collecting data. In the IGT, four decks of cards labeled A′, B′, C′ or D′ are displayed on the computer screen. The subject is required to select one card at a time from one of the four decks. When the subject selects a card, a message is displayed on the screen indicating the amount of money the subject has won or lost. Choosing a card can result in an immediate reward (the immediate reward is higher in decks A′ and B′ relative to Decks C′ and D′). As the game progresses, there are also unpredictable losses associated with each deck. Total losses are on average higher in decks A′ and B′ relative to decks C′ and D′, thus creating a conflict in each choice, i.e., decks A′ and B′ are disadvantageous in the long-term (even though they bring higher immediate reward), whereas decks C′ and D′ are advantageous in the long-term (i.e., the long-term losses are smaller than the short-term gains, thus yielding a net profit). Net decision-making scores are obtained by subtracting the total number of selections from the disadvantageous decks (A′ and B′) from the total number selections from the advantageous decks (C′ and D′). Thus, positive numbers reflect good decisions, while negative numbers reflect bad decisions.

### fMRI protocol

Participants lay supine on a scanner bed and viewed visual stimuli back-projected onto a screen through a mirror built into the head coil. The IGT was written in Matlab (Mathworks) based on Psychtoolbox (www.psychtoolbox.org). Participants were given instructions on the IGT. Details of these instructions have been published previously (Bechara et al., 2000). We used an event-related design of the IGT which was described in a recent paper (Koritzky et al., 2013). Each trial of the IGT includes two phases: a decision phase and a feedback phase. In the decision phase, participants were requested to select a card from four Decks (A′, B′, C′ or D′) by pressing the corresponding button when a message (“Pick a Card”) was displayed at the center of screen. In the feedback phase, a message was shown to inform the participants how much money they won or lost based on their choice of cards. The time for the responses to be made in the decision phase was between 3 s and 7 s. The mean was 4 s since this interval varied randomly between trials. At the feedback stage, participants were informed how much money they won or lost by their selected card. The feedback phase last for 3 s. If the trial is a win-only trial (i.e., no loss), the feedback (“you win $X”) was displayed for 1.5 s, followed by a 1.5 s blank screen. If the trial is a win-but-loss trial, the win feedback (“you win $X”) was displayed for 1.5 s, followed by a 1.5 s display of the loss feedback (“but you also lose $X”). The mean length of the inter-trial interval was 2 s with variation from 1.1 s to 3.2 s. The design was optimized with an in-house program to maximize efficiency. There were total 100 trials and lasted for 15 min.

fMRI was acquired in the Dana and David Dornsife Cognitive Neuroscience Imaging Center at the USC with a 3T Siemens MAGNETOM Tim/Trio scanner. Z-SAGA sequence with PACE (Prospective Acquisition Correction) was used for functional scan to collect blood oxygen level-dependent (BOLD) signals. This specific sequence is dedicated to reduce signal loss in the prefrontal and orbitofrontal areas, with the following scanning parameters: TR/TE = 2000/25 ms; flip angle = 90°; 64 × 64 matrix size with resolution 3 × 3 mm^2^. Thirty-one 3.5-mm axial slices were used to cover the whole cerebral cortex and most of the cerebellum with no gap. The anatomical T1-weighted structural scan was done using an MPRAGE sequence (TR/TE/TI = 2530/3.1/800 ms; flip angle 10°; 208 sagittal slices; 256 × 256 matrix size with spatial resolution as 1 × 1 × 1 mm^3^).

### fMRI analysis

FEAT (fMRI Expert Analysis Tool, part of FSL package, www.fmrib.ox.ac.uk/fsl) was used for image preprocessing and statistical analysis. Standard preprocessing procedures were performed including brain extraction, image realignment, smooth (5 mm FWHM Gaussian kernel), and temporal filtering (100 s cut-off). A two-step registration procedure was used whereby EPI images were first registered to the MPRAGE structural image, and then into standard MNI space, using affine transformations (Jenkinson and Smith, 2001). Registration from MPRAGE structural image to standard space was further refined using FNIRT non-linear registration. Statistical analyses were performed in the native image space, with the statistical maps normalized to the standard space prior to higher-level analysis.

The data were modeled at the first level using a general linear model within FSL's FILM module. To examine brain activations related to decision making, two types of events were modeled: (1) decision-making stage, and (2) feedback stage. In this paper, we were particularly interested in the BOLD responses related to the decision-making phase (i.e., the deck selection of the IGT). The event onsets were convolved with a canonical hemodynamic response function (HRF, double-gamma) to generate the regressors used in the GLM. Temporal derivatives were included as covariates of no interest to improve statistical sensitivity. Null events were not explicitly modeled, and therefore constituted an implicit baseline. Missing trials were modeled separately as a nuisance variable. The six movement parameters were also included as covariates in the first-level general linear model.

Higher level random-effect model was tested for group activation in decision making stages (i.e., decision making stage VS baseline) in particular using FMRIB's Local Analysis of Mixed Effect stage 1 only (Beckmann et al., 2003; Woolrich et al., 2004) with automatic outlier detection (Woolrich, 2008). Unless otherwise noted, group images were thresholded using cluster detection statistics, with a height threshold of *Z* > 2.3 and a cluster probability of *p* < 0.05, corrected for whole-brain multiple comparisons based on Gaussian Random Field Theory (GRFT).

To test the correlation between brain activation in the decision making phase of IGT and dietary intake, region of Interests (ROI) were created from clusters of voxels with significant activation in the voxelwise analyses. Brain activation (% signal change) in these regions when making decisions was extracted using a method suggested by Mumford (http://mumford.fmripower.org/perchange_guide.pdf). Robust regression was used to minimize the impact of outliers in the behavioral data, using iteratively reweighted least squares implemented in the robustfit command in the MATLAB Statistics Toolbox (Tom et al., 2007). Reported *r*-values reflect (non-robust) Pearson product-moment correlation values, whereas the reported *p*-values and regression lines are based on the robust regression results (Tom et al., 2007).

## Results

### Behavior results

#### Demographic variables

Participants in the study fell within the normal range of the body mass index (BMI). Average BMI was 21.88 ± 1.62, with a range of 19.1–25. IQ scores were all within a normal range (118.29 ± 8.6, range = 103–132). Participants reported 2.57 ± 1.88 on the hunger rating scale (1-not at all hungry; 10-extremely hungry), reflecting the fact that they were being evaluated in a non-food deprived state. With regard to dietary intake, participants reported consuming 2.95 ± 2.15 servings/day of vegetables and 1.0 ± 0.84 servings/day of fatty and sugary snacks. Participants reported consuming significantly more vegetables than snacks in their daily life [*T*_(23)_ = 3.52, *p* < 0.01]. No age or gender differences were observed on consumption of vegetables or snacks, BMI, IQ, or IGT net scores and hunger ratings.

#### Partial correlations

Table 1 shows partial correlations among the following variable measures: vegetables, snacks, BMI, IQ, the IGT net scores, and hunger ratings after controlling for age and gender. Vegetable consumption did not correlate with consumption of snacks (*r* = −0.01, *p* > 0.05). Although these relationships were not statistically significant, vegetable and snack consumption were negatively and positively correlated with BMI (*r* = −0.19, *r* = 0.21, respectively). Moreover, none of the variables were significantly correlated with the IGT net scores. Finally, the more vegetables the participants consumed in their daily life, the higher their self-reported hunger rating prior to the fMRI session (*r* = 0.43, *p* < 0.05, corrected for multiple comparison).

**Table 1:** **Partial correlations among vegetables, snacks, BMI, IQ, SOPT and the IGT net scores after controlling for age and gender**.

**Measures**	**2**	**3**	**4**	**5**	**6**
1. Vegetables	−0.01	−0.19	0.30	0.16	0.43^*^
2. Snacks		0.21	−0.15	0.13	0.03
3. BMI			−0.02	0.08	−0.1
4. IQ				−0.01	−0.11
5. IGT net scores					−0.22
6. Hungry rating					

Results of two-tailed significance tests are denoted by superscripts.

* *P < 0.05, IGT = Iowa Gambling Task*.

#### IGT performance

**Figure 1:**
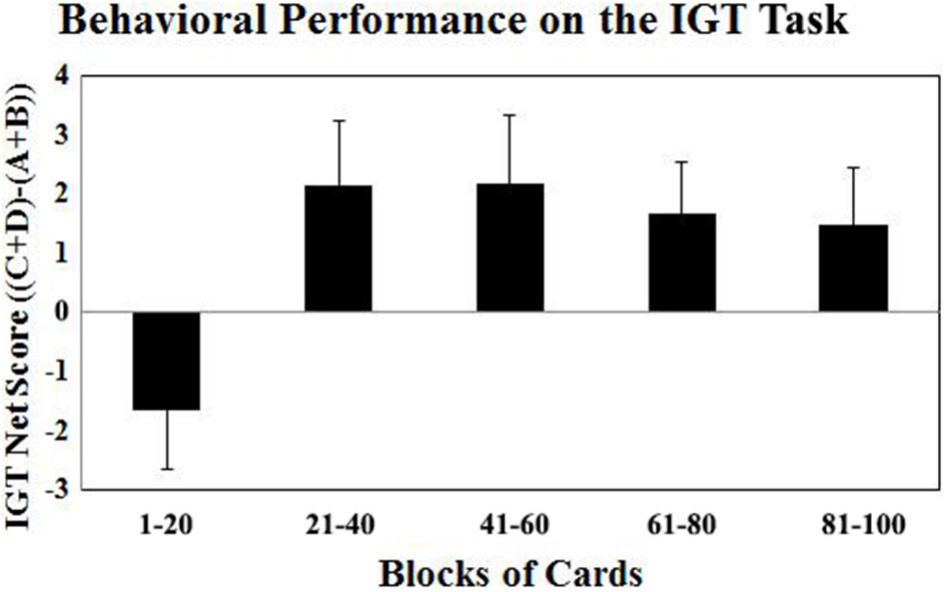
**The Iowa Gambling Task net scores ((C′ + D′) – (A′ + B′)) across five blocks of 20 cards expressed as mean ± SE**. Positive net scores reflect advantageous (non-impaired performance) while negative net scores reflect disadvantageous (impaired) performance.

The fMRI optimized version of the IGT task involved 100 trials (or 100 card selections). The trials are divided into five blocks of 20 trials each. In each block, the number of selections from Decks A′ and B′ (the disadvantageous decks) and the number of selections from Decks C′ and D′ (the advantageous decks) are counted and a net score for each block ((C′ + D′) – (A′ + B′)) is obtained. A net score above zero implies that participants are selecting cards advantageously, and a net score below zero implies disadvantageous selection. The behavioral results revealed a significant effect of block after the Greenhouse-Geisser adjustment [*F*_(3.6, 81.7)_ = 5.98; *P* < 0.001]. As shown in Figure 1, the participants in this study showed normal learning as the task progressed. They gradually switched their preferences toward the advantageous decks (C′ and D′) and away from the disadvantageous decks (A′ and B′), as reflected by increasingly positive net scores.

### Neuroimaging results

#### IGT activity during the decision stage

**Figure 2:**
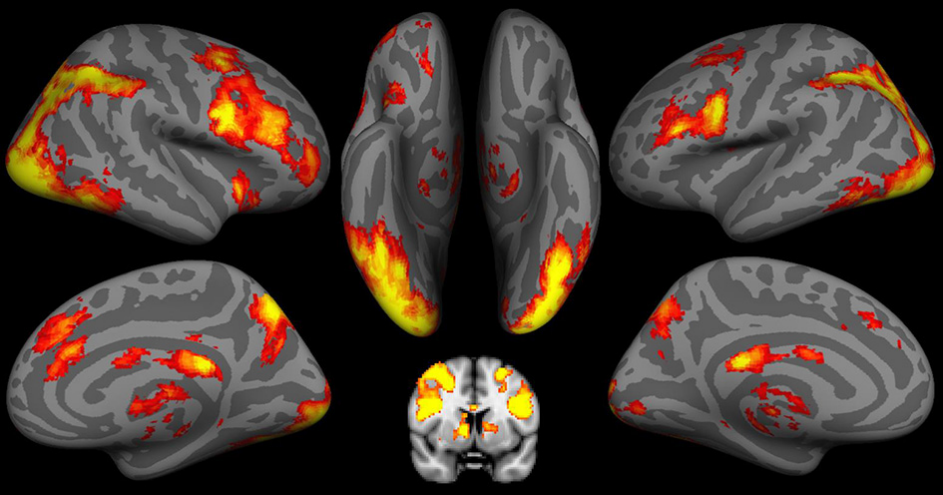
**fMRI results of the Iowa Gambling Task (IGT) during the decision stage**. Both the impulsive system, including the bilateral putamen/caudate, and the reflective system including the bilateral dorsoateral prefrontal cortex (DLPFC), ventromedial prefrontal cortex (VMPFC), and anterior cingulate cortex (ACC) are involved in the decision stage of the IGT. Activation in IGT also includes insula and visual cortex.

As shown in Figure 2 and Table 2, during the decision stage, the IGT activated brain regions belonging to both the impulsive system (namely the right amygdala and ventral striatum) and the reflective system (namely the VMPFC and dorsolateral prefrontal cortex (DLPFC), and anterior cingulate cortex (ACC). The IGT also elicited activity in the “urge/craving” system, namely the insular cortex. Activity was also observed in additional neural regions (e.g., temporal cortex, post-central cortex and visual cortex), but there were no *a priori* hypotheses regarding the roles of these brain regions in the behaviors under the current study.

**Table 2:** **Brain activity of the Iowa Gambling Task during the decision stage**.

**L/R**	**Brain regions**	**N of voxels**	**MNI coordinates**			**Z**
			**x**	**y**	**z**	
L/R	Visual cortex	17663	−6	−96	−2	6.05
R	Frontal pole/VMPFC/DLPFC	2277	42	0	58	5.03
L/R	Thalamus/Brain stem/Ventral striatum	1321	−6	−24	6	5.03
L	Frontal pole/VMPFC/DLPFC	1217	−36	52	16	4.82
L/R	ACC	1002	4	26	32	5.13
L	SPL/SMG	569	−30	−54	36	4.36
L/R	PCC	478	−2	−26	24	5.50
L	Post-central cortex	300	−58	−20	46	4.20
L	Temporal cortex	302	−62	−20	16	4.78
R	Amygdala/Ventral striatum	216	28	−2	−10	3.61
L	Hippocampus	151	−18	−28	−10	4.27
L	Insula	110	−38	2	0	3.45
R	Insula	89	42	14	−4	3.73

*VMPFC: Ventromedial Prefrontal Cortex; DLPFC: Dorsolateral Prefrontal Cortex; SPL: Superior Parietal Lobe; SMG: Suparamarginal Cortex; ACC: Anterior Cingulate Cortex; PCC: Posterior Cingulate Cortex*.

#### Correlations between brain activity and eating behaviors

**Figure 3:**
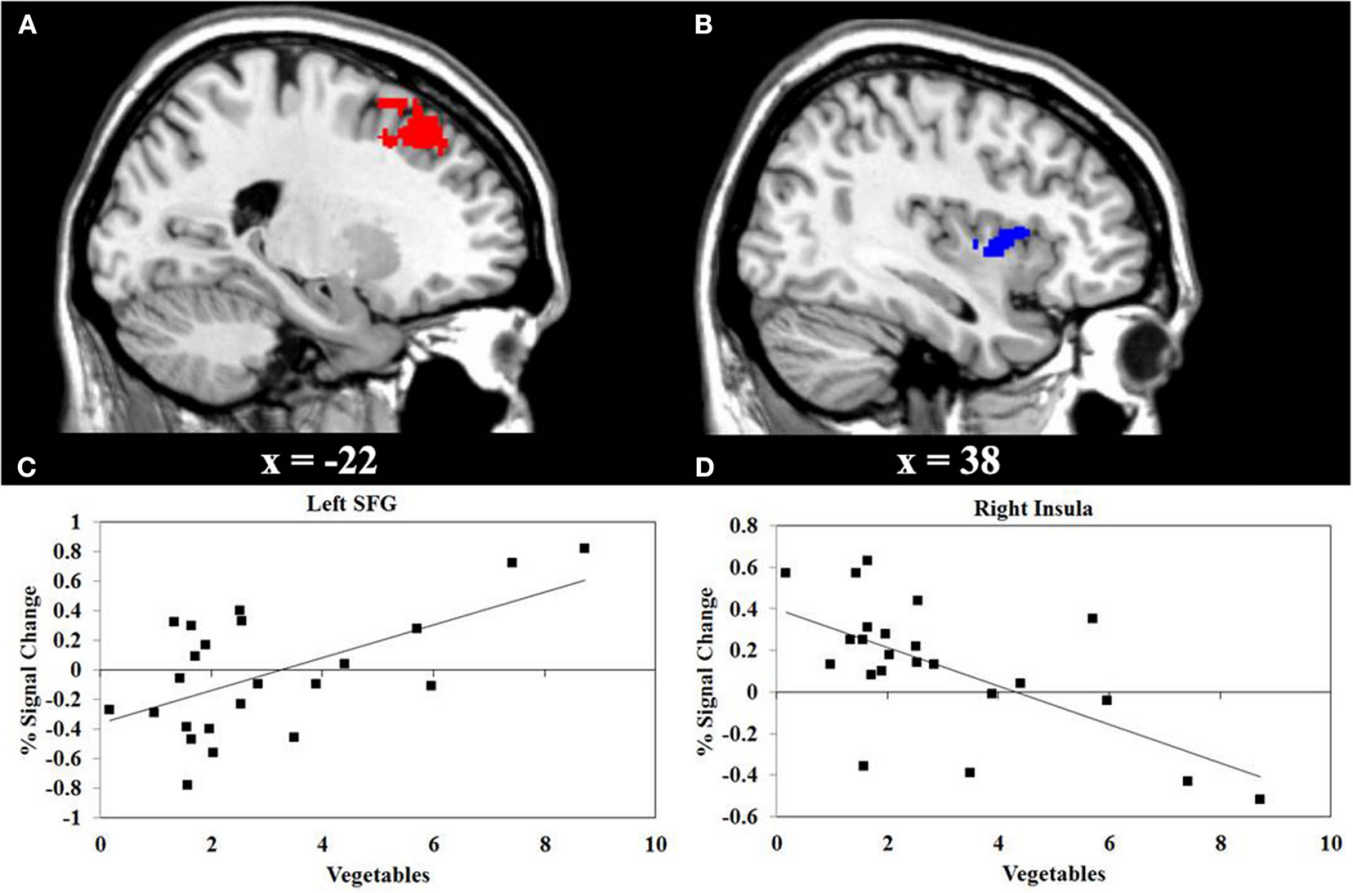
**Functional MRI correlation of vegetable consumption and IGT activity during decision stage of the Iowa Gambling Task (IGT). (A)** Regions show a significant positive correlation (red) between vegetable consumption and the left superior frontal gyrus (SFG) activation. **(B)** Regions show significant negative correlation (blue) between vegetable consumption and the right insula. **(C, D)** Scatterplots of correlations between vegetable consumption and the averaged covariance of the parameter estimates in the left SFG and right insular cortex, respectively.

**Figure 4:**
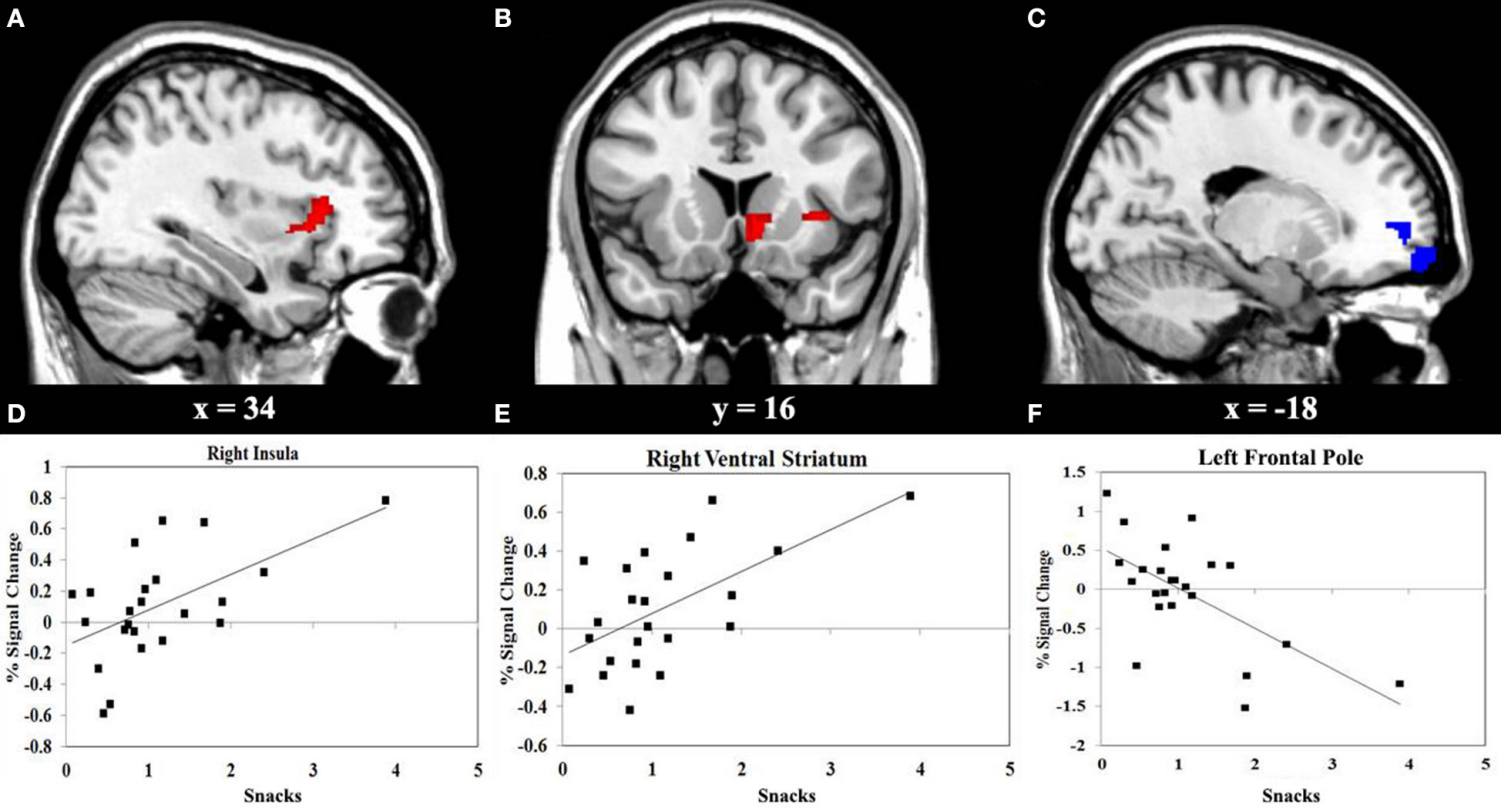
**Functional MRI correlation of snack consumption and the Iowa Gambling Task (IGT) activity during decision stage. (A)** Regions show significant positive correlation (red) between snack consumption and right insular cortex activation. **(B)** Regions show significant positive correlation (red) between snack consumption and right ventral striatum activation. **(C)** Regions show significant negative correlation (blue) between snack consumption and left frontal pole. **(D, E, F)** Scatterplots of correlations between snack consumption and the averaged covariance of parameter estimates in the right insular cortex, right ventral striatum and left frontal pole, respectively.

We performed a correlation analyses between the consumption of vegetables or snacks, and the BOLD response elicited by IGT performance in the decision stage. The results shown in Figure 3 reveals that higher consumption of vegetables correlates with higher activity in the left superior frontal gyrus (SFG) (*r* = 0.55, *P* < 0.01), and with lower activity in the right insula (*r* = −0.66, *P* < 0.001). Figure 4 reveals that higher snack consumption correlates with lower activity in the left frontal pole (*r* = −0.63, *P* < 0.001), and with higher activity in the right ventral striatum (*r* = 0.60, *P* < 0.01) and right insular cortex (*r* = 0.56, *P* < 0.01).

## Discussion

In the current study, IGT performance elicited neural activity in neural systems hypothesized to play key roles in complex decision-making: (1) neural regions belonging to the so-called “reflective system” concerned with impulse control and self-control, namely the VMPFC, the DLPFC, as well as the ACC in both hemispheres; (2) neural regions belonging to the so-called “impulsive system” concerned with reward and habit behaviors, namely the striatum in both hemispheres; and (3) neural systems implicated in the processing of interoceptive signals and their translation into what may subjectively become experienced as an urge, namely the insula in both hemispheres. Moreover, higher consumption of vegetables positively correlated with activity in the left superior frontal gyrus (SFG) (i.e., a component of the reflective system), but negatively correlated with activity in the right insular cortex. In contrast, high consumption of snacks negatively correlated with activity in the left frontal pole (a part of the reflective system), but positively correlated with activity in the right ventral striatum and right insula cortex.

These results are consistent with several behavioral studies showing that poor decision-making scores measured by the IGT are found in obese, patients with binge eating disorders, and overweight adolescents (Pignatti et al., 2006; Brogan et al., 2010; Verdejo-Garcia et al., 2010; Danner et al., 2012; Fagundo et al., 2012). They are also consistent with previous reports that performance on the IGT was related to the magnitude of weight loss in a diet-induced weight loss intervention in overweight women (Witbracht et al., 2012). The brain regions implicated in this study are also consistent with several previous studies on food (high vs. low calorie), weight (obese vs. average weight), and activity in neural regions (Killgore et al., 2003; Pelchat et al., 2004; DelParigi et al., 2005, 2006; Killgore and Yurgelun-Todd, 2005; Davis et al., 2007, 2010; Stice et al., 2008; Small, 2009; Batterink et al., 2010; Ng et al., 2011; He et al., 2014b). The unique contribution of our current study is the use of a neural framework that assigns multiple neural regions to functionally specialized neural systems involved in behavioral decisions (Naqvi and Bechara, 2010; Noel et al., 2013). More importantly, our current study examines the dynamics among these neural systems (i.e., hyperactivity in one system, but hypoactivity in another). The examination of these dynamics is especially significant in terms of devising therapeutic strategies.

High consumption of high-calorie snacks in real-life correlated with higher activity in the ventral striatum. The ventral striatum has long been known for its role in various types of reward, including food reward (Demos et al., 2012; Mehta et al., 2012). Animal studies indicate that direct pharmacological activation of the ventral striatum increases preferentially the intake of foods high in fat and sugar, even in animals fed beyond apparent satiety (Petrovich et al., 2002; Kelley, 2004). In humans, several lines of evidence suggest that high calorie food may induce greater incentive values in obese individuals compared to normal controls (Volkow et al., 2012; Tomasi and Volkow, 2013). Behavioral studies also show that compared to their normal controls, overweight children indicate that high calorie food (pizza and snack food) is more reinforcing (Temple et al., 2008). Thus, our current findings are consistent with this long line of studies in both animals and humans.

A unique aspect of the current study is that we used a monetary reward in order to engage the neural systems sub-serving decision-making instead of food reward. The results indicate that the observed changed dynamics between these neural systems apply not only to food, but to reward in general, including monetary reward. This is quite consistent with the conceptualization about a common currency for reward that relates to dopamine, especially that associated with the ventral striatum (McClure et al., 2004b). Many studies have shown that this region is similarly engaged by food as well as monetary cues. For instance, increased ventral striatal activity (reflecting increased dopamine) potentiated the rewarding effects of food as well as the association between food cues and the feeling of pleasure associated with food consumption (Smith and Robbins, 2013). Also the anticipation of food (as opposed the experience of food) is rewarding and it is associated with increased ventral striatal activity (that presumably reflects increased dopamine release) (Smith and Robbins, 2013). Even the numerous behavioral studies in humans that suggested that obese individuals are hyper-responsive to food cues in a wide range of assessments (Braet and Crombez, 2003; Halford et al., 2004), and the behavioral studies in both healthy and overweight populations suggesting that personality traits of reward drive predict food craving, overeating, and relative body weight (Davis and Woodside, 2002; Bulik et al., 2003; Davis et al., 2004). are all considered as consistent with the constructs that increased reward sensitivity is linked to a biologically-based personality trait regulated by mesocorticolimbic dopamine systems (Cohen et al., 2005; Evans et al., 2006). Indeed the increased neuronal activity elicited by fatty food cues in the ventral striatum predicted the macronutrient choice at an *ad libitum* buffet, i.e., greater ventral striatum activity predicted the choice of food items with higher fat content (Mehta et al., 2012). This ventral striatal activity also predicted weight gain 6 months later (Demos et al., 2012). In parallel, these same striatal regions responsive to food cues have also been shown to respond in a similar manner to monetary reward (Breiter and Rosen, 1999; Breiter et al., 2001), thus supporting the notion that altered dynamics between these neural systems may be general, and not specific to food reward.

Higher right insular activity correlated with more snack, but less vegetable, consumption in real life. Given the hypothesized role of the insular cortex in translating interoceptive signals into what one may subjectively experience as a feeling of desire, anticipation or urge (Naqvi and Bechara, 2009; Noel et al., 2013), we suggest that engaging the insula system increases the urge or craving for high calorie food by (1) exacerbating activity within the striatal (impulsive) system, and (2) weakening activity of the prefrontal (reflective) system [e.g., see (Noel et al., 2013)]. This suggestion is consistent with studies showing that activity within the insular cortex is associated with food craving (Pelchat et al., 2004). Finally, our study revealed a role for prefrontal regions (parts of the reflective system) in the inhibitory control of some high calorie food items, consistent with several previous studies suggesting a role for the SFG in introspection, self-judgments, and the subjective rating of self-awareness (Goldberg et al., 2006). Goldberg et al. proposed that the left SFG is involved in allowing the individual to reflect upon sensory experiences, to judge their possible significance to the self, and to allow the individual to report about the occurrence of his sensory experience to the outside world (Goldberg et al., 2006). Others implicated the frontal pole area (Broadmann 10) in insight into one's future and the planning of future actions (McClure et al., 2004a; Fellows and Farah, 2005; D'Argembeau et al., 2008; Koritzky et al., 2013). These studies are quite consistent with our early conceptualization on the role of these regions in what we called a “reflective” system in the context of other rewards, namely drugs (e.g., Bechara, 2005). However, the novel contribution of the current study is the examination of the dynamics between multiple neural systems (e.g., hypoactivity in the reflective system combined with hyperactivity in the striatal and insula systems in response to high calorie food).

Although our early conceptualization about an imbalance between an impulsive and reflective system was initially discussed in the context of drug reward (Bechara, 2005), a similar conceptualization argued that eating disorders and obesity may be associated with a mismatch between the impulsive and reflective systems (Gearhardt et al., 2011; Brooks et al., 2013; García-García et al., 2013). Our study is very consistent with these earlier reports, except that we now show that this imbalance also applies to normal people who are not necessarily diagnosed with obesity or eating disorder. Since our study was cross-sectional, we are not able to make inferences about whether the differences in the neural substrates of decision-making reflect the cause or effect of real-life food consumption. It is likely that activities of these brain systems mediate the development of our eating behaviors. This is pertinent to the argument made by some researchers that we should emphasize the importance of focusing on high-risk food substances (and their potential to alter specific brain systems) rather than high-risk people, which has tended to be the focus of most research to date (Gearhardt and Brownell, 2013). An emphasis on such future research could provide an insight on the neural basis and related cognitive and behavioral interventions that help weight management and prevent obesity and other eating disorders (Paolini et al., 2012; Gearhardt and Brownell, 2013).

Finally, we note that the IGT is a task that taps into the brain mechanisms sub-serving decision-making, but it only involves abstract money/points as a reward, as opposed to food reward. Thus, the task itself does not ask subjects to consume real food, nor to view images of food while in the scanner. As such, the current study using the IGT could potentially be deemed as non-ecological valid, and thus limit the generalization of our results. However, we argue the opposite in that the use of the IGT had several important advantages. First, the use of food related executive function tasks (e.g., go/no go tasks with food stimuli) has been reported multiple times in the literature and yielding consistent results (He et al., 2014b). Second, even structural volumetric measures of ROIs within the so-called “reflective system” showed consistent negative correlations with BMI, independent of using any tasks that involve food images (He et al., 2014a). Hence, the current results using the IGT, which is a complex task that taxes the functions of all three neural systems hypothesized to be engaged in addiction (Li et al., 2010; Xiao et al., 2013), suggest that the relatively poor ability to delay gratification from high calorie food reward is not specific to food reward, but it generalizes to other rewards (and in this case it is monetary reward). These findings are significant as they support the notion that the process leading to overweight and obesity is one that is reflected by a relative imbalance in neural systems implicated in addictive behaviors, and also decision-making in general.

### Conflict of interest statement

The authors declare that the research was conducted in the absence of any commercial or financial relationships that could be construed as a potential conflict of interest.
